# A Potential Role for MMPs during the Formation of Non-Neurogenic Placodes

**DOI:** 10.3390/jdb6030020

**Published:** 2018-07-26

**Authors:** Paige M. Drake, Tamara A. Franz-Odendaal

**Affiliations:** 1 Department of Medical Neuroscience, Dalhousie University, 5850 College Street, Halifax, NS B3H 4R2, Canada; paige.drake@dal.ca; 2 Department of Biology, Mount Saint Vincent University, 166 Bedford Highway, Halifax, NS B3M 2J6, Canada

**Keywords:** extracellular matrix, morphogens, embryo, matrix metalloproteases, tooth, lens, mammary gland, feather, hair, β-catenin, patterning

## Abstract

The formation of non-neurogenic placodes is critical prior to the development of several epithelial derivatives (e.g., feathers, teeth, etc.) and their development frequently involves morphogenetic proteins (or morphogens). Matrix metalloproteinases (MMPs) are important enzymes involved in extracellular matrix remodeling, and recent research has shown that the extracellular matrix (ECM) can modulate morphogen diffusion and cell behaviors. This review summarizes the known roles of MMPs during the development of non-neurogenic structures that involve a placodal stage. Specifically, we discuss feather, hair, tooth, mammary gland and lens development. This review highlights the potential critical role MMPs may play during placode formation in these systems.

## 1. MMPs in the ECM

The extracellular matrix (ECM) is the non-cellular component of all tissues. It not only provides the physical scaffolding for cellular constituents but also initiates crucial biochemical and biomechanical cues that are essential for tissue morphogenesis, differentiation and homeostasis. The ECM is mainly composed of proteoglycans (e.g., decorin, hyaluronic acid, heparin sulfate proteoglycans (HSPGs)) and fibrous proteins (e.g., collagen, elastin, fibronectin, laminin). The ECM is a highly dynamic structure that is constantly remodeled either enzymatically or non-enzymatically. Matrix metalloproteinases (MMPs) are the main enzymes involved in ECM degradation. Their activity is usually low in normal conditions but is activated during matrix remodeling and repair. During tissue homeostasis, MMP activity is counterbalanced by tissue inhibitors of MMPs (TIMPs), other enzymes, and growth factors that direct the remodeling processes [1,2,3]. MMPs may be produced as soluble or cell membrane-anchored proteinases. If secreted, they are activated in the ECM space via proteolytic cleavage, often by another MMP, or via oxidation through reactive oxygen species. Membrane-bound MMPs are anchored to cell membranes; either via a transmembrane domain or a GPI linkage. 29 MMPs are identified in sea urchins [4] and a mere two in *Drosophila* [5]. The number of MMP genes in vertebrates is 23 in mice [6], 24 in humans [7], 25 in zebrafish [8], and 26 in frog [9]. However, due to the teleost genome duplication there is not a straightforward relationship between these suites of MMPs; thus, zebrafish are missing some MMPs but have duplicates of the others [10]. MMPs also cleave with a wide range of substrate specificities and can collectively cleave all ECM proteins (Table 1).

**Figure 1:**
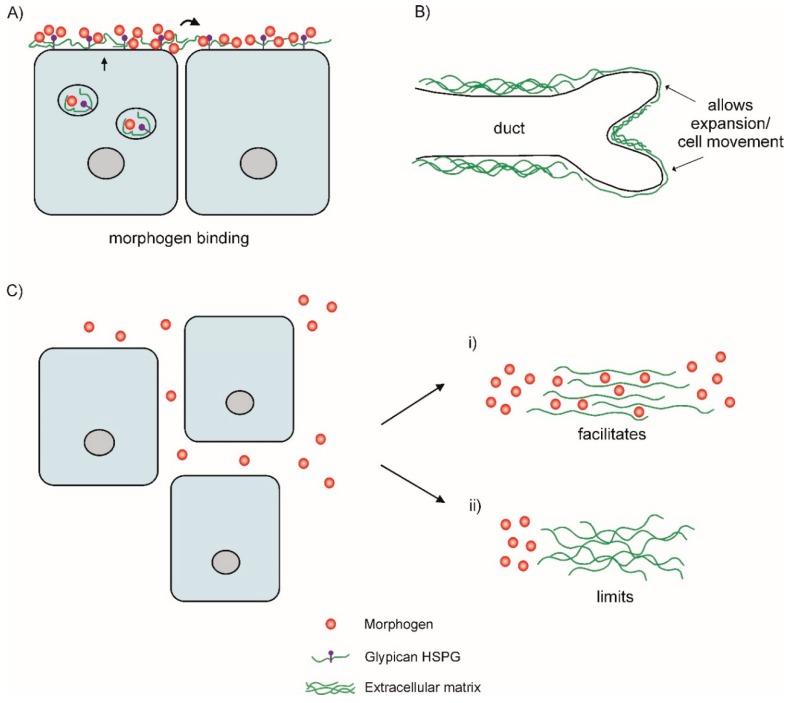
Mechanisms by which the extracellular matrix (ECM) affects cell movements and both morphogen binding and diffusion. MMPs are important enzymes in remodeling the ECM for these various purposes. (**A**) Morphogens can bind to the extracellular matrix via proteins, such as glypican heparan sulfate proteoglycans (HSPGs) and cell receptors. (**B**) Areas of thick ECM restrict cell movement, whereas a thin ECM allows for tissue expansion necessary in processes like branching morphogenesis. (**C**) The ECM can modify morphogen diffusion rates by either facilitating or limiting morphogen diffusion through the ECM. Modified from References [17,19].

In the words of Rozario and DeSimone [12], “the ECM can be thought of as a morphogenetic code that is interpreted by cells that come into contact with it”. This interpretation by the ECM usually occurs via receptors and has a profound influence on the cell’s behavior (e.g., movement, polarity, proliferation, differentiation, etc.). Specifically, during embryogenesis, the ECM provides the structural integrity needed for cell movements, modifies the diffusion of morphogens, and provides binding sites for a number of cell surface receptors (including integrins and syndecans) (Figure 1). There are indeed many examples of extracellular secreted morphogens interacting with the ECM during development—the ECM is thus considered a highway for long-range facilitated diffusion of signaling molecules [13,14,15,16,17,18].

Secreted morphogens that play critical roles during development include members of the Wnt, Hedgehog (Hh), transforming growth factor-beta (TGF-β) and fibroblast growth factor (FGF) families. By binding to the heparan sulfate proteoglycans (HSPGs) in the ECM, for example, the diffusion of morphogens along the cell surfaces of receiving cells is restricted (reviewed in Yan et al., 2009). In other systems, HSPGs provide a signaling platform for morphogens by enabling them to interact with other important components (e.g., co-receptors, other secreted proteins). FGFs bind to FGFRs in a heparin sulfate-dependent fashion such that FGF-FGFR binding and dimerization can be modulated by heparin sulfate. Furthermore, heparin sulfate can dictate the radius of FGF signaling by defining the diffusion potentials of FGFs through the ECM [20].

Thus, ECMs interact with morphogens and morphogens are key to placode formation. Given also that the ECM is constantly remodeled, and that this remodeling occurs largely via MMPs, we therefore sought to determine whether MMPs play a role in the development of non-neurogenic placodes. We determined that while much is known about the morphogens involved in placode formation, fewer studies mention the ECM molecules involved and extremely few mention specific role(s) for MMPs. Most studies investigating MMPs in these systems have examined MMPs during development of the placode derivative structures (hairs, feathers, etc.), i.e., the later stages of development. We summarize this information here and highlight the MMPs that should be further investigated in each system based on the known ECM molecules involved in each system.

## 2. Placodes

**Figure 2:**
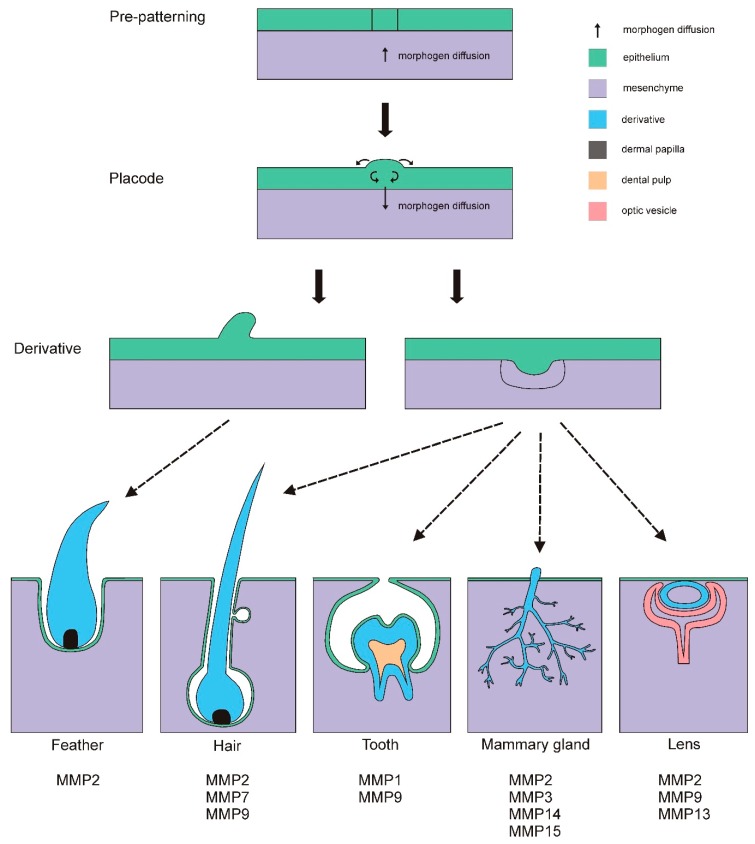
Placode-derived structures begin development with pre-patterning of the epithelium, which involves morphogen diffusion from the mesenchyme to the epithelium. Next, a thickening of the epithelium forms a placode structure. Following the formation of a placode, morphogen signaling from the epithelium down to the mesenchyme leads to the formation of a derivative structure. Some MMPs are involved in some of these processes (see text for details).

Placodes are distinct thickened regions of the epithelium that are critical for the development of many cutaneous structures, such as feathers, teeth, hair, etc. (Figure 2). These placodes arise after the epithelium receives molecular signals from the underlying mesenchyme and/or surrounding epithelium. How this homogenous field of epithelial cells undergoes patterning to give rise to distinct placodal regions is a central question in developmental biology. In some systems, a field of cells responds to a gradient of inducing morphogens while in others inducing signals act over very short distances to produce an all-or-nothing response [21]. Lateral inhibition or gap junction communication can also play a role in patterning a distinct cell field (such as a placodal region) to emerge. In some systems, turing-mechanisms of diffusing activators and inhibitors set up the placodal field. Below, we discuss the current literature on MMPs during placodal development in vertebrates with the goal of pointing out gaps in our current understanding of their potentially very important role in pattern formation.

### 2.1. Feather and Hair Development

During feather and hair morphogenesis, a series of reciprocal epithelial-mesenchymal interactions result in epidermal placodes to form overlying mesenchymal (or dermal) cell aggregations. The first signal originates from the mesenchyme to induce the epithelial thickening or placode, which then signals back to induce the dermal condensation. Spatial rearrangements of dermal cells involve at least two distinct ECM components—fibronectin and interstitial collagen—and leads to their aggregation underneath each epidermal placode [22]. Members of the canonical Wnt signaling pathway (β-catenin and Lef-1) are present in the skin prior to hair formation and are elevated in the placodes as they form [23]. Specifically, Wnt 10a and 10b, as well as their Frizzled (Fz) receptors Fz 1 and Fz 10, are found in epithelial placodes giving rise to hairs. During feather formation, several Wnts (Wnt-1, -3a, -7a) and β-catenin are expressed at high levels in the prospective feather placodal epithelium, with Wnt 5a expressed in the interplacodal epithelium and Wnt 11 restricted to the interplacodal dermis [23]. DKK4, an inhibitor of canonical Wnt signaling, is expressed transiently in the early stage epithelial placodes of hairs [23]. Although no MMPs have been shown to be involved during hair placode formation, MMP9 and MMP2 are present at later stages of hair follicle morphogenesis [24] and MMP7 (or matrilysin) is present in the keratinocytes of the hair placode [25]. MMP2 (and also TIMP2) is co-localized to the feather epithelium and distal mesenchyme in the short-bud stage of feather formation, which is the stage immediately after the placode stage [26]. Through MMP inhibition studies, these researchers further showed that MMP activity is essential in the early stages of feather development.

A number of MMP substrates are also associated with the placode stage of feather development. Tenascin was identified as one of the earliest adhesion molecules present in feather placodes [27]. Both fibronectin and interstitial collagen are also known to be involved in the formation of feather placodes—fibronectin for cell adhesion and collagen fibrous bundles to support the center of future feather rudiments [22]. It has been suggested that collagen bundles provide a pathway for dermal cells to migrate beneath the developing feather placodes—a process that is hypothesized to be disrupted in areas of featherless skin produced by hydrocortisone treatment [22,28]. A heterogeneous pattern of fibronectin and collagens I and III are associated with normal development of feather placodes [29], whereas an even distribution of these components occurs in the skin of the scaleless (−/−) mutant chicken and in hydrocortisone-treated skin [30]. The involvement of fibronectin, tenascin and collagens during feather placode development, suggests that several MMPs may be involved in remodeling these matrices to support placode formation. Specifically, MMPs 1–16, MMP19, MMPs 24–26. Further research is needed to elucidate the complete role of MMPs during feather placode development—research to date has likely only touched the surface of this understanding.

### 2.2. Mammary Gland Development

In mice, mammary gland development begins during embryogenesis between embryonic day (E) 10.5 and 18.5, when mammary cell lines migrate from the limb buds to the ventral side of the embryo. Five pairs of mammary placodes then develop in the ectoderm. In the early placode stage, the Wnt inhibitor, DKK4, is transiently expressed [23]. These placodes subsequently invaginate into the mesenchyme to form mammary buds, where they are surrounded by a primary layer of mesenchyme and located above an adipocyte-rich compartment called the mammary fat pad [31]. The primary mesenchyme develops into an area of dense connective tissue associated with the nipple. The mammary buds begin sprouting mammary ducts during ductal morphogenesis, which infiltrate the underlying fat pad to form initial ductal trees. Postnatally, these ducts remain dormant until puberty, after which hormones stimulate the epithelium of the ducts (terminal end buds) to proliferate within the mammary fat pads—a process termed branching morphogenesis [32]. In adulthood, the glands undergo further structural modification during pregnancy, lactation and involution.

Several members of the MMP protease family have been associated with the tissue remodeling involved in various stages of mammary gland development. Although this research has yet to be conducted at the placode stage, much of it has focused on the role of MMPs during branching morphogenesis. Both proteolytic and non-proteolytic regions of MMP14 have been shown to be important for proper epithelial branching and invasion [33,34]. Despite both MMP14 and MMP15 having been implicated with branching morphogenesis using in vitro models, a recent study by Feinberg et al. [35] demonstrated in vivo that MMP14 null and MMP15 null mice lacking these proteases had little to no effect on ductal branching. However, transcriptome and histological analyses did reveal that MMP14 promotes adipogenesis of white fat, while MMP15 suppresses brown fat production [35]. Additional research is required to better elucidate these subtle effects of MMP14 and -15 on mammary gland development.

Less subtle are the effects of MMP2 and MMP3 on branching morphogenesis. MMP2 likely works with other genes activated by bone morphogenetic protein 4 (BMP4) in the mesenchyme to initiate ductal morphogenesis at the bud stage [36]. Initially, MMP2 promotes invasion of the terminal end buds and supports cell survival during branching morphogenesis, while later it represses the rate of lateral branching [37]. Overexpression of MMP3 causes supernumerary lateral branching, increased reactive oxygen species, and promotes the development of mammary cancer [38,39]. Kessenbrock et al. [40] later reported that hyperplastic epithelial growth is a result of binding and inhibiting Wnt5a activity, and that overexpressing MMP3 increases mammary stem cell activity. Despite these roles for both MMP2 and MMP3, in vivo studies have also demonstrated that ductal branching proceeds normally in MMP2 and MMP3 null mice [35,37].

Several ECM molecules are involved in mammary gland branching morphogenesis. Namely: Fibronectin, laminin, and collagens. While there is an abundance of information on this later stage of mammary gland development, it remains to be seen whether or not MMPs are involved in the earlier (placodal) stages of mammary gland development.

### 2.3. Tooth Development

Tooth development in mice begins with a placodal thickening of the oral epithelium that becomes a bud surrounded by condensed mesenchyme. During the early placode stage, DKK4, a Wnt signaling inhibitor, is transiently expressed [23]. This is followed by the cap and bell stages, during which the tooth crown is formed. Tooth cusps form after folding at the epithelial-mesenchymal junction with predentin secreted by odontoblasts and enamel by ameloblasts.

There are four main MMPs that are expressed throughout the bud, cap, and bell stages of tooth development in rats: MMP1, -2, -3 and -9 [41]. These four MMPs are expressed throughout cells of the epithelium and mesenchyme at the bud stage [41,42]. At the cap stage, MMP1 is more strongly expressed in the underlying condensing mesenchyme than in the epithelium, suggesting that it plays a more continuous role in ECM remodeling needed for mesenchymal condensation and invagination [41]. Notably, MMP3 expression is localized to the enamel knot during the cap stage, indicating a possible role in ECM turnover associated with the degradation of this structure [41]. In the early bell stage, all four MMPs are expressed in the inner enamel epithelium, basement membrane, and pre-odontoblast cells, with negligible expression in the dental papilla [41,43,44]. It is suggested that ECM remodeling occurs at this epithelial-mesenchymal interface in order to create the space needed for odontoblast and ameloblast differentiation [41]. In the late bell stage, a gradient of MMP expression is observed in the dental papilla, where stronger expression is correlated with newly differentiated odontoblasts [41]. Therefore, all four MMPs may play a role in modifying the predentine matrix prior to mineral deposition [41]. Additionally, Sahlburg et al. [42] found that the MMPs that they investigated (MMP2 and MMP9) during tooth development were colocalized with their respective TIMPs. This finding lends support to the general concept that remodeling is a balance between protease and inhibitor activity.

The basement membrane is an important ECM tissue that serves as the interface of the epithelial-mesenchymal interactions that facilitate tooth development [44]. Once dentin secretion has begun, however, the basement membrane is removed to allow direct interaction between odontoblasts and ameloblasts [45]. Because type IV collagen is the main constituent of the basement membrane, MMP2 and MMP9 are thought to be the chief collagenases responsible for basement membrane degradation [43,44]. MMP2 and MMP9 degrade similar ECM components and are both expressed predominantly in the mesenchyme during early tooth development—however, they are differentially expressed in later stages of tooth development [42]. MMP2, coupled with the TIMP2 inhibitor, likely participate in basement membrane degradation prior to the secretion of dentin matrix [42], whereas Heikinheimo & Salo [44] found only low levels of MMP9 expression during tooth development. MMP9 and the TIMP3 inhibitor are co-localized in osteoclasts that are associated with ECM turnover leading to tooth eruption [42]. Postnatally, there is also evidence that MMP9 may play a role in amelogenin processing during enamel formation in mice [46]. In rat incisors, MMP3 is reported in the odontoblast and ameloblast cells that secrete dentine and enamel, respectively [47,48]. MMP20 is one of two main proteases secreted by ameloblasts during enamel formation where it functions to replace organic matrix material to make way for the deposition of a hard, non-porous enamel layer [49]. In addition to developmental and remodeling contexts, it has been argued that many of the MMP enzymes can also play a role in pathological contexts as evidenced by their enhanced expression associated with dental caries and inflammation of pulp and periapical tissues [50].

The entire process of tooth development certainly involves extensive ECM remodeling by MMPs, other proteases, and TIMPs. However, MMP activity, if any, at the initial placode stages of tooth development has yet to be determined.

### 2.4. Lens Development

In mice, eye development begins at embryonic day (E) 9 with a bilateral set of optic vesicles. The optic vesicles adhere to the surface ectoderm of the head, which leads to thickening of the ectoderm to form the lens placode. The lens placode then invaginates with the optic vesicles, to form the lens pit and optic cup. These two structures separate - the lens pit turns into the lens vesicle which becomes the lens, while the optic cup gives rise to the retina, ciliary epithelia, iris epithelia, and retinal pigment epithelium.

There has been little in the way of MMP-related research conducted in the context of lens development, let alone at the placode stage. Walter et al. [51], in an attempt to identify additional genes involved in the lens development of *Xenopus laevis*, identified MMP13 expression in the anterior central nervous system and presumptive lens epithelium between developmental stages 24–35. Lens placodes in this species develop at stage 26, thus, these authors hypothesized that MMP13 may be important for lens placode formation in this species. Furthermore, fibronectin, a substrate degraded by several MMPs, has decreased expression in the lens placode of Pax6 knockout mice and deleting it results in failure of the lens placode to form and invaginate [52]. Thus, this MMP substrate is likely important at the placode stage of lens development and the MMPs that cleave it should be further investigated (Table 1).

Other research has investigated the effect of MMP2 and MMP9 expression on cataract formation. For example, elevated levels of TGF-β induce cataracts through epithelial-mesenchymal transition of lens epithelial cells into myofibroblasts, the cells that secrete alpha smooth muscle actin, thus creating the reduced lens clarity characteristic of cataracts [53,54]. Additionally, treating lens cells with TGF-ß increases the expression of MMP2 and MMP9 [55,56]. Pino [57] confirmed that the increased MMP9 expression, which lies upstream of MMP2 [58] is induced by TGF-β and causes the dissolution of E-cadherin/β-catenin complexes that are responsible for the epithelial-mesenchymal transition that ultimately forms cataracts. Thus, MMPs have a role in lens epithelial cell differentiation.

Recent research by Huang et al. [52] highlighted the need to investigate the mechanism of lens placode formation. Although this study lacks any specific mention of MMPs, it does argue that the leading hypothesis behind formation of the lens placode is a matrix-mediated process. Their work supports the “restricted expansion hypothesis” of placode formation, whereby adhesion between the ECM and surface ectoderm, as opposed to expansion, allows for cell proliferation to create an epithelial thickening that ultimately results in lens placode formation [52,59]. Their results also demonstrate that Pax6 and fibronectin-1 are required for proper placode formation, and suggest that placodes provide the mechanical conditions sufficient for invagination of the lens placode into the lens pit [52]. Given how crucial the ECM environment is to lens placode formation and invagination, there is the need for more studies investigating the specific roles of MMPs during these processes.

### 2.5. Other Placode Systems

Although less studied than the systems reviewed above, MMPs have also been studied in the context of the salivary gland, otic, and lateral line systems. In the developing salivary gland, both MMP14 and MMP15 cleave collagens during branching morphogenesis [60]. In otic placodes, MMP15 is expressed during but not after otic placode and vesicle formation [61,62]. Otic placodes are sites of significant basement membrane remodeling, with laminin turnover having specific involvement in otic vesicle formation [63]. Interestingly, laminin is one of the main substrates of MMP15 [64] suggesting a contribution of this enzyme towards remodeling activity within otic placodes. There are several other MMPs that also have laminin as a substrate that may also be involved in otic placode formation (Table 1). The lateral line system in fish and aquatic amphibians, a mechanosensory organ used to detect changes in water movement and pressure, arises from six placodal cell populations [65,66,67,68,69,70]. Invadolysin, part of a metalloprotease family separate from MMPs, disrupts angiogenesis and cell migration into neuromast subpopulations during lateral line development [71]. Crawford et al. [72] also showed that MMP25b is expressed in the developing lateral line ganglia in zebrafish. Thus, although there is some research highlighting the role of MMPs in other placodal systems, this is very limited and warrants rigorous further investigation.

## 3. Conclusions and Future Directions

Research addressing the role of matrix metalloproteases during placode development is lacking. Most of the studies to date regarding the presence and/or role of these enzymes during derivative development takes place at later stages—such as branching morphogenesis in salivary and mammary glands; bud, cap and bell stages of tooth development; the short-bud stage of feather development; and the later stages of hair follicle morphogenesis. Thus, while several MMP substrates have been studied at various stages of derivative development, the importance of ECM remodeling as the placode initiates and progresses to subsequent stages of development calls for a closer look at the involvement of MMPs during these critical stages of development.

Of all the MMPs that have been characterized to date, some MMPs seem to be more involved than others when it comes to ECM remodeling in placode-derived systems. Namely, MMP2, -3, -9, -14, and -15 are most commonly mentioned in the current literature. It is also important to keep in mind the model organisms that these findings are based on (the mouse *Mus musculus*, chick *Gallus gallus*, and frog *Xenopus laevis*). Future research should thus target the MMPs whose substrates have been shown to be important in placode development (Table 1). Furthermore, Several ECM molecules (including several fibrous proteins—fibronectin, tenascin) and some MMPs (e.g., MMP9) are directly regulated by miRNAs in development [73]. Micro-RNAs (miRNAs) can control ECM compositions and thus the behavior of cells that reside in that ECM. Whether or not miRNAs play a role during non-neurogenic placode formation is unknown at present but is a potential avenue for future research directions.

Additionally, β-catenin signaling is upstream of MMP expression in both physiological and pathological tissue morphogenesis [74,75,76,77]. This is particularly interesting, since β-catenin is a key pre-patterning molecule expressed at the earliest stages of placode formation in a number of systems (including feather, scutes, teeth, etc.) [78,79,80,81,82]. Therefore, further research could investigate the nature of interactions between MMPs and β-catenin in the context of placode development. Despite the variety of research conducted to date, additional research is needed to fully explore the importance of MMPs during placode formation.

## Figures and Tables

**Table 1:** Matrix metalloproteinases (MMPs), their known extracellular matrix substrates, and the placodal systems in which their substrates have been studied. Modified from Reference [11].

MMP	Substrate	Placodal Systems
1	Collagens (I, II, III, VII, VIII, X and XI), entactin, proteoglycans, ovostatin, MMP2, MMP9, pro-MMP9, fibronectin, vitronectin, laminin, tenascin, aggrecan, link protein, myelin basic protein, versican	Tooth, feather, otic, lens
2	Collagens (I, II, III, IV, V, VII, X and XI), gelatin, elastin, fibronectin, vitronectin, laminin, entactin, tenascin, SPARC, aggrecan, link protein, galectin-3, versican, decorin, myelin basic protein	Hair, feather, mammary gland, tooth, lens, otic
3	Collagens (III, IV, V, VII, IX, X and XI), gelatin, aggrecan, laminin, elastin, casein, osteonectin, fibronectin, ovostatin, entactin, plasminogen, pro-MMP9, vitronectin, tenascin, SPARC, link protein, decorin, myelin basic protein, perlecan, versican, fibulin	Mammary gland, tooth, feather, otic, lens
7	Collagens (I and IV, gelatin, fibronectin, laminin, elastin, transferrin, casein, vitronectin, SPARC, aggrecan, decorin, versican, fibulin, myelin basic protein	Hair, feather, lens
8	Collagens (I, II and III), fibronectin, proteoglycans, aggrecan	feather, lens
9	Collagens (IV, V, VII, X and XIV), gelatin, elastin, fibronectin, vitronectin, laminin, SPARC, aggrecan, link protein, galectin-3, versican, decorin, myelin basic protein	Hair, feather, tooth, lens, otic
10	Collagens (III, IV, V), gelatin, casein, elastin, fibronectin, aggrecan, link protein	feather, lens
11	Collagen IV, laminin, elastin, fibronectin, casein, proteoglycans	Feather, otic, lens
12	Collagen IV, elastin, gelatin, casein, fibronectin, vitronectin, laminin, entactin, fibrinogen	Feather, otic, lens
13	Collagens (I, II, III, IV, VI, IX, X and XIV), large tenascin-C, plasminogen, aggrecan, fibronectin, SPARC, gelatin, perlecan MMP9	Lens, feather
14	Collagens (I, II and III), gelatin, fibronectin, laminin, vitronectin, entactin, pro-MMP2, aggrecan, perlecan,	Mammary gland, salivary gland, feather, otic, lens
15	Fibronectin, gelatin, vitronectin, entactin, laminin, pro-MMP2, tenascin, perlecan	Mammary gland, salivary gland, otic, feather, lens
16	Collagen III, gelatin, casein, fibronectin, pro-MMP2, laminin, vitronectin	Feather, otic, lens
17	Gelatin, fibrinogen, pro-MMP2	
18	Collagen I	
19	Collagens (I and IV), gelatin, fibronectin, laminin, entactin, large tenascin-C, fibronectin	Feather, otic, lens
20	Amelogenin, aggrecan	
21	Gelatin, casein	
23 (A, B)	Gelatin	
24	Fibronectin, pro-MMP2, proteoglycans, gelatin	Feather, lens
25	Collagen IV, gelatin, fibronectin, pro-MMP2, pro-MMPp	Feather, lens
26	Collagen IV, fibrinogen, fibronectin, gelatin, pro-MMP9, vitronectin	feather
27	Gelatin, casein	
28	Casein	
